# Supplementary material for: Generalising uncertainty improves accuracy and safety of deep learning analytics applied to oncology
Source: Sci Rep. 2023 May 6;13:7395. doi: 10.1038/s41598-023-31126-5 (PMC10164181; doi:10.1038/s41598-023-31126-5)
Supplement: Supplementary file 1 — Supplementary Information. [file 41598_2023_31126_MOESM1_ESM.docx]

**Supplementary Information**

**Generalising uncertainty improves accuracy and safety of deep learning analytics applied to oncology**

MacDonald S. et al., 2022

[S1 Supplementary Methods 2](#_Toc126304004)

[S1.1 Data profile, preprocessing, and stratification 2](#_Toc126304005)

[S1.1.1 Data Profile 2](#_Toc126304006)

[S1.1.2 RNAseq data preprocessing 3](#_Toc126304007)

[S1.1.3 Sample label preprocessing 4](#_Toc126304008)

[S1.2 Selection of evaluation metrics 4](#_Toc126304009)

[S2 Supplementary Tables 4](#_Toc126304010)

[S3 Supplementary Figures 6](#_Toc126304011)

[S4 Application of ADP to the CIFAR-10 dataset 17](#_Toc126304012)

[S5 Supplementary Theory - Distributional Shift Model Effects 17](#_Toc126304013)

[S5.1 DL loss functions depend on IID data 17](#_Toc126304014)

[S5.2 Out-of-domain data leads to ‘silent catastrophic failure’ 18](#_Toc126304015)

[S6 Supplementary References 18](#_Toc126304016)

#

#

#

# S1 Supplementary Methods

## S1.1 Data profile, preprocessing, and stratification

### S1.1.1 Data Profile

Training was performed on a publicly available cancer dataset - The Cancer Genome Atlas (TCGA) [1]. Test data came from the TCGA metastatic samples [2], Met500 dataset [3] and a custom collection of studies, i.e. our Internal Custom Data (ICD) [4]–[12]. A common exclusion criteria for all datasets (TCGA, ICD, Met500) was if sample IDs were duplicates, or if they belonged to cancer types with 10 (or less) samples. If duplicate sample IDs had different gene expression information, it was the duplicate with a higher amount of zero-valued read counts which was excluded. These independent datasets consisted of tumour samples from primary cancers, metastatic cancers, as well as cancer classes that were visible during training , termed ‘Seen’, or not, termed ‘Unseen’ (Supplementary Table 1).

TCGA gene expression data were downloaded as HTSeq counts (ENCODE v22) from an online data repository - National Cancer Institute Genomic Data Commons (GDC; downloaded on 23^rd^ Mar 2020). A total of 11,093 sample files were downloaded. Samples were excluded if clinical data were incomplete or if the tissue was normal (i.e. non-cancerous). Approximately 15% of the primary tumour samples were withheld by stratified random sampling to serve as in-sample validation (n=1,434) while the remaining 85% serve as training set (n=8,202). Both training and validation partitions of TCGA data spanned over 32 (primary) tumour types. A held-out TCGA test dataset (n=392) hosted 11 metastatic cancer types, all of which were present in the training and validation partitions.

ICD gene expression data were collated from multiple sources reported in the corresponding original studies [4]–[12]. ICD data consisted of 461 samples, spanning seven cancer types (five ‘Seen’ - primary and metastatic, and two ‘Unseen’). ICD data were preprocessed using the TCGA RNAseq pipeline to produce HTSeq counts. Briefly, sequencing reads were aligned to GRCh38 with STAR (2.5.2a) and gene expression counts were produced by HTSeq (0.11.2) using ENCODE v22 to match TCGA data.

Met500 gene expression data were downloaded as FPKM normalised counts (ENCODE v23, downloaded 10th Oct 2020) from the University of California Santa Cruz (UCSC [website](https://xenabrowser.net/datapages/?cohort=MET500%20(expression%20centric)) link; [3].

### S1.1.2 RNAseq data preprocessing

The preprocessing procedure was done in a manner that is reproducible in the setting where models are deployed from development (i.e. training on TCGA primary) to production (i.e. testing on TCGA metastatic, ICD, and Met500). The TCGA whole transcriptome consists of 60,483 transcripts identified by unique Ensembl gene accession numbers (Ensembl ID) based on the GENCODE v22 assembly (ref). The transcriptome was subsequently reduced to the general Ensembl ID (by removing the version number) shared across all training and test datasets (i.e. TCGA, ICD, and Met500).

Genes from the TCGA primary dataset where all samples had zero expression were excluded from all datasets (TCGA, ICD and Met500), which yielded 18,945 remaining genes. Read counts were then converted into transcripts per million (TPM) normalised gene expression by first normalising for gene length followed by normalisation of sequencing depth.


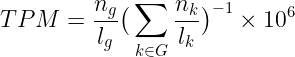


**Equation 1.** Where *n_g_* is the number of reads for gene *g*, *l_g_* is the normalised gene length, and *G* is the set of all genes.

Gene lengths were calculated as the sum of all exon lengths for each gene provided in the GENCODE v22 GTF annotation file. TCGA and ICD datasets were aligned. Met500 raw read counts data were not available and thus required transforming FPKM to to TPM counts.The FPKM counts were converted to TPM format by first approximating read count proportions by multiplying the FPKM values with the Ensembl gene lengths derived from GENCODE v23 assembly, followed by applying the TPM normalisation procedure. TPM counts were then log2-transformed. The minimum and maximum values for each of the genes within TCGA primary data formed the min-max parameters used for scaling all other datasets between zero and one.

### S1.1.3 Sample label preprocessing

Cancer type labels for all datasets (TCGA, Met500, and IDC) were reviewed and aligned to the OncoTree convention [13], where possible, with a pathologist’s guidance (Supplementary Table 4). The cancer types with 10 or less samples were excluded from the TCGA dataset after OncoTree label alignment.

## S1.2 Selection of evaluation metrics

In this study , we chose to restrict ourselves to the micro-F1 for evaluation of predictive quality to avoid deviating from the primary focus of addressing shift-induced overconfidence about individual samples. We believe this restriction is justified, as micro-F1 is relatable to the F1-AUC and ADP metrics which both depend on micro-F1 and uncertainty’s correlation with the error-rate (the micro-F1’s inverse). Nonetheless, micro-F1 can be misleading for imbalanced classification problems, especially when the training inter- and sub-class distributions are different from testing datasets [14]. Hence, for predictive interpretations, metrics such as the macro-F1, or Matthew’s Correlation Coefficient (MCC), where classes contribute equally, as well as the Area Under the Receiving Operating Characteristic Curve (ROC-AUC), or Area Under the Precision-Recall Curve (PR-AUC) may have been supportive.

# S2 Supplementary Tables

**Supplementary Table 2. Hyperparameter Configuration.**

| **Hyperparameter** | **Setting** |
| --- | --- |
| Batch Size | 216 |
| Max number of epochs | 200 |
| Min-max range | [0 ,1] |
| Number of Monte Carlo Samples | 50 (in-training), 250 (in-test) |
| Ensemble size | 10 |
| Hidden layer neuron count (i.e. layer widths) | [1024, 1024, 1024, 1024] |
| Weight initialisation method | ‘Kaiming normal’ |
| Non-linear activation | Mish |
| Dropout probability | 0.4 |
| Weight decay (L2 regularisation) | 5e-3 |
| Optimiser | Adam with AMS Grad |
| Learning rate | 1e-4 |
| Patience | 20 (23 for Resnet)* |
| Precision | 32 |
| Singular value bound (Spectral norm) | 1.1 (hidden layers), 8.5 (input layer), 1.1^3 (output layer) |
| Batchnorm momentum | 0.01 (hidden layers), 0.1 (first input layer) |

* Resnet was trained with a patience of 23 (not 20) to help train time match BNNs to control for accuracy and number of epochs.

**Supplementary Table 3. Minimum sample size for each model when estimating ADP.**

| **Model** | **Min Sample Size (IID)** | **Min Sample Size (OOD)** |
| --- | --- | --- |
| Resnet | 1328 | 431 |
| MCD | 1333 | 399 |
| Ensemble | 1330 | 391 |
| Bilipschitz | 1319 | 367 |

#

# S3 Supplementary Figures


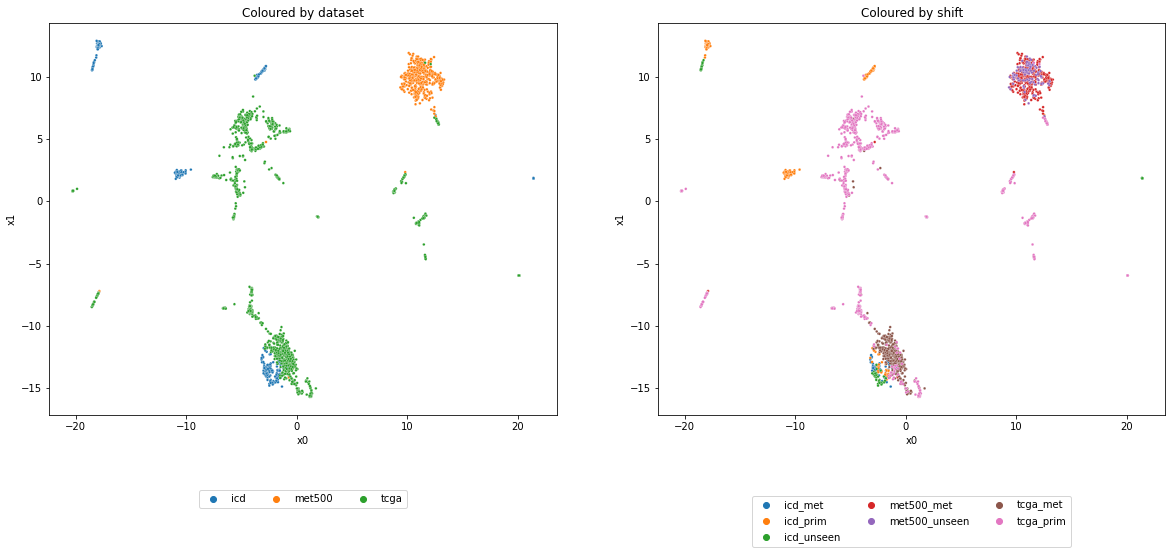


**Supplementary Figure 1. Two-dimensional projection of three independent datasets.** Data inputs for all ICD and Met500 datasets, as well as a subset of TCGA primary data used for validation (35 samples from each class) and all TCGA metastatic data. Left plot is colour coded by the datasets. The right plot is colour coded by the strata defined by the unique shift with the pink ‘tcga_prim’ labels indicating training and validation (i.e. development) IID data. Dimensionality reduction performed with pacMAP [15].

**
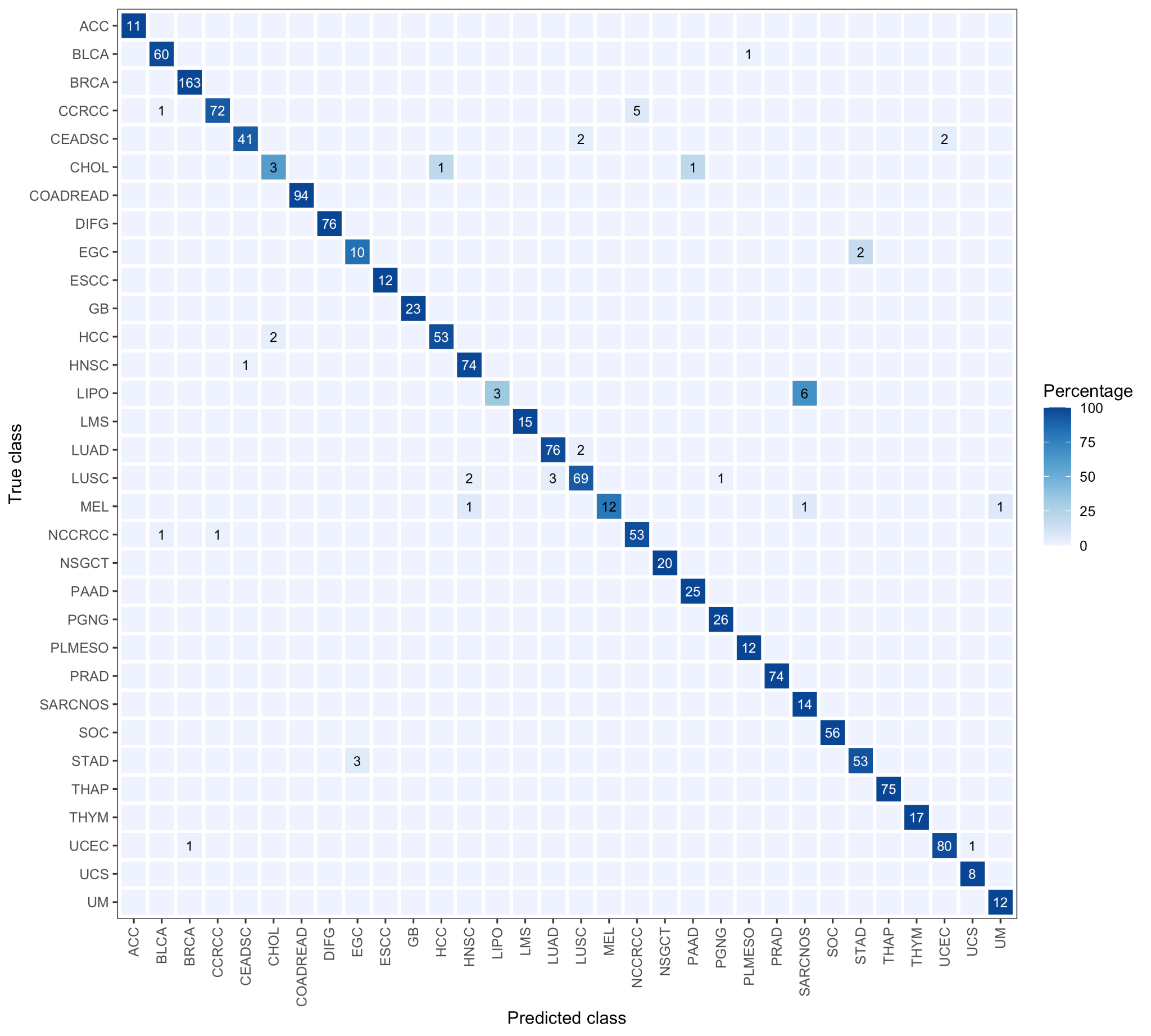
**

**Supplementary Figure 2. Confusion matrix of Resnet predictions for IID validation data.**

Each row corresponds to the true cancer type, and each column corresponds to the cancer type prediction. Coloured scale indicates the percentage of all samples in a particular class, while the numbers correspond to the number of samples predicted as a particular class.

**
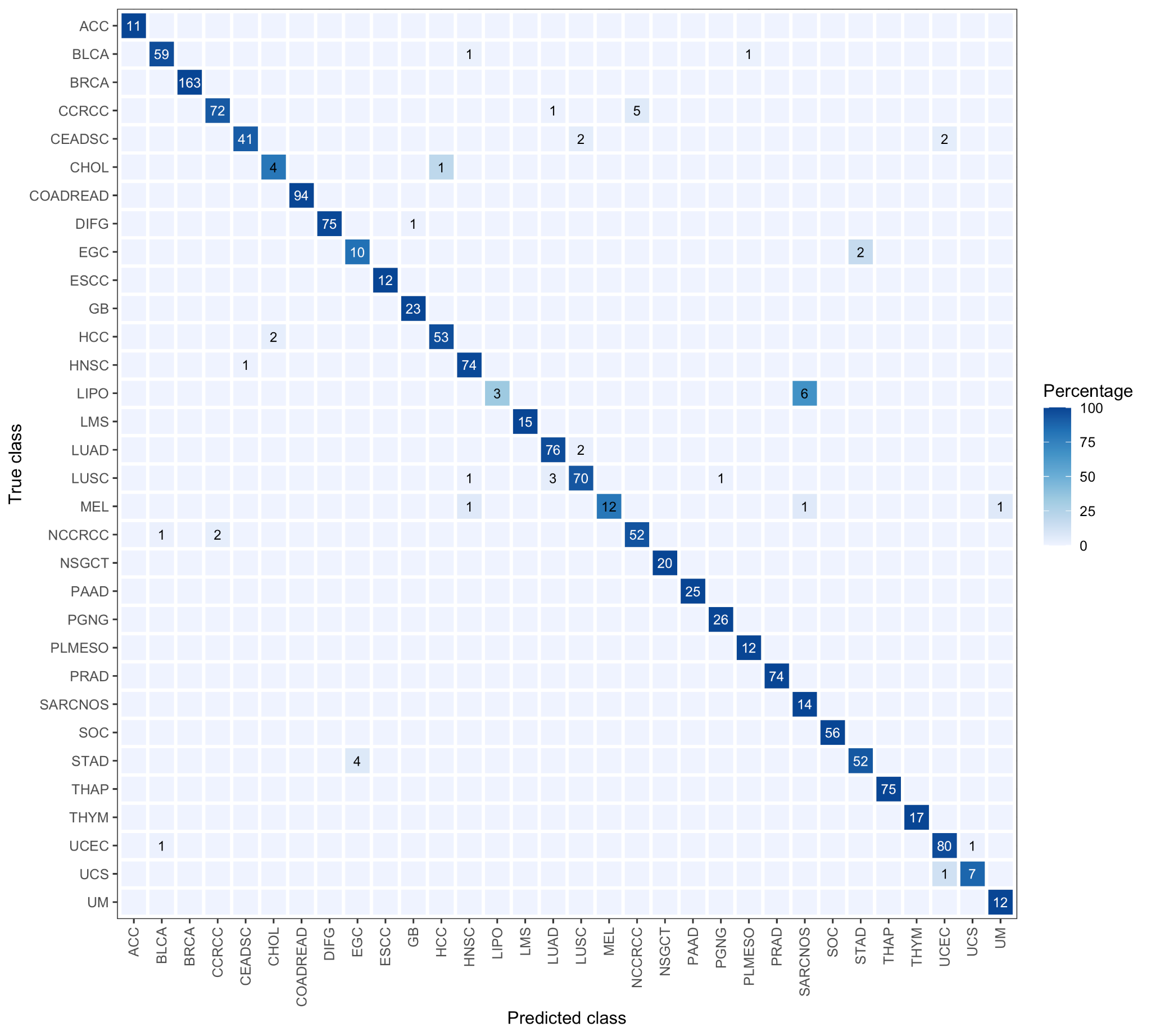
**

**Supplementary Figure 3. Confusion matrix of MCD predictions for IID validation data.** Each row corresponds to the true cancer type, and each column corresponds to the cancer type prediction. Coloured scale indicates the percentage of all samples in a particular class, while the numbers correspond to the number of samples predicted as a particular class.


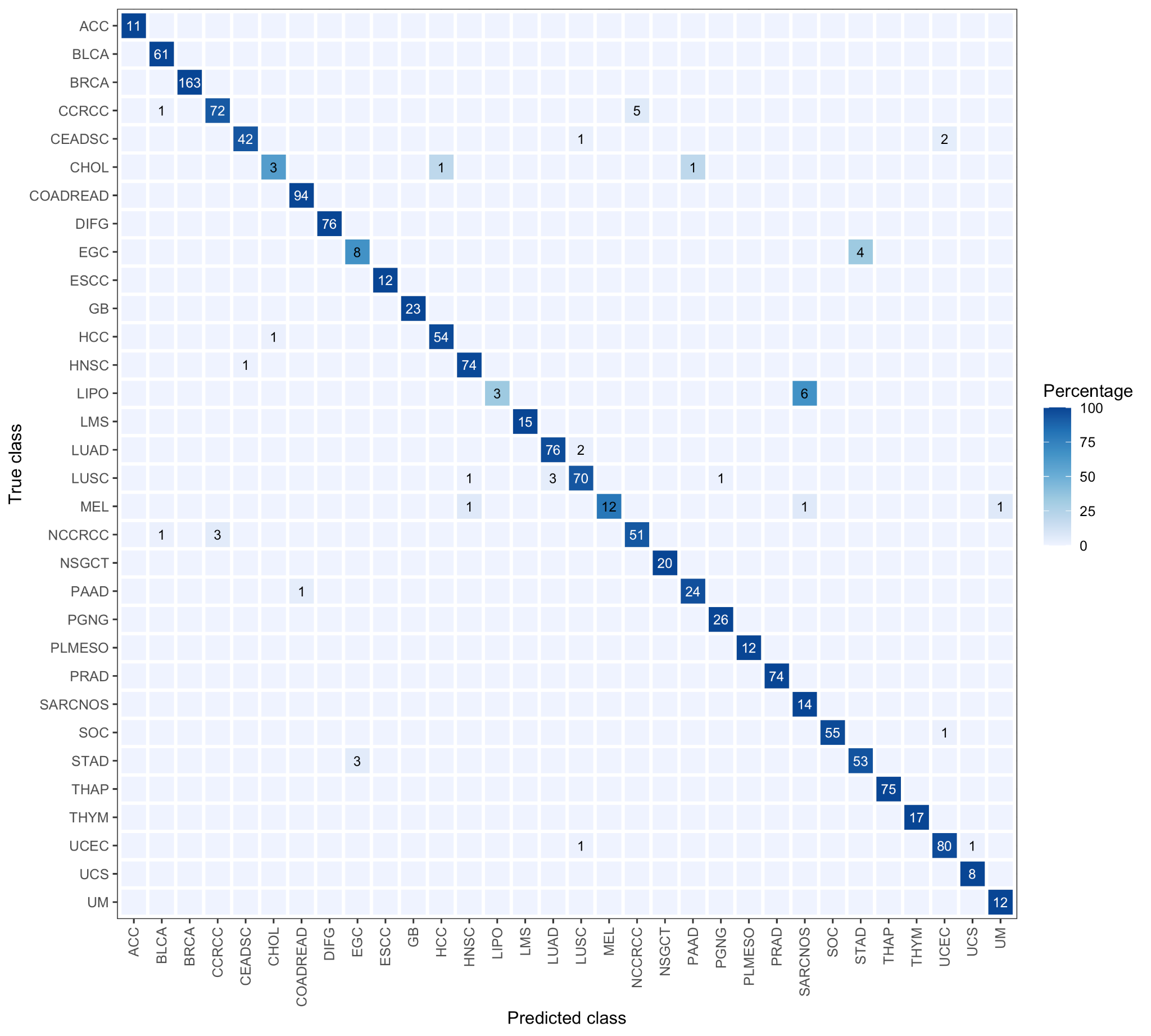


**Supplementary Figure 4. Confusion matrix of Bilipschitz predictions for IID validation data.** Each row corresponds to the true cancer type, and each column corresponds to the cancer type prediction. Coloured scale indicates the percentage of all samples in a particular class, while the numbers correspond to the number of samples predicted as a particular class.


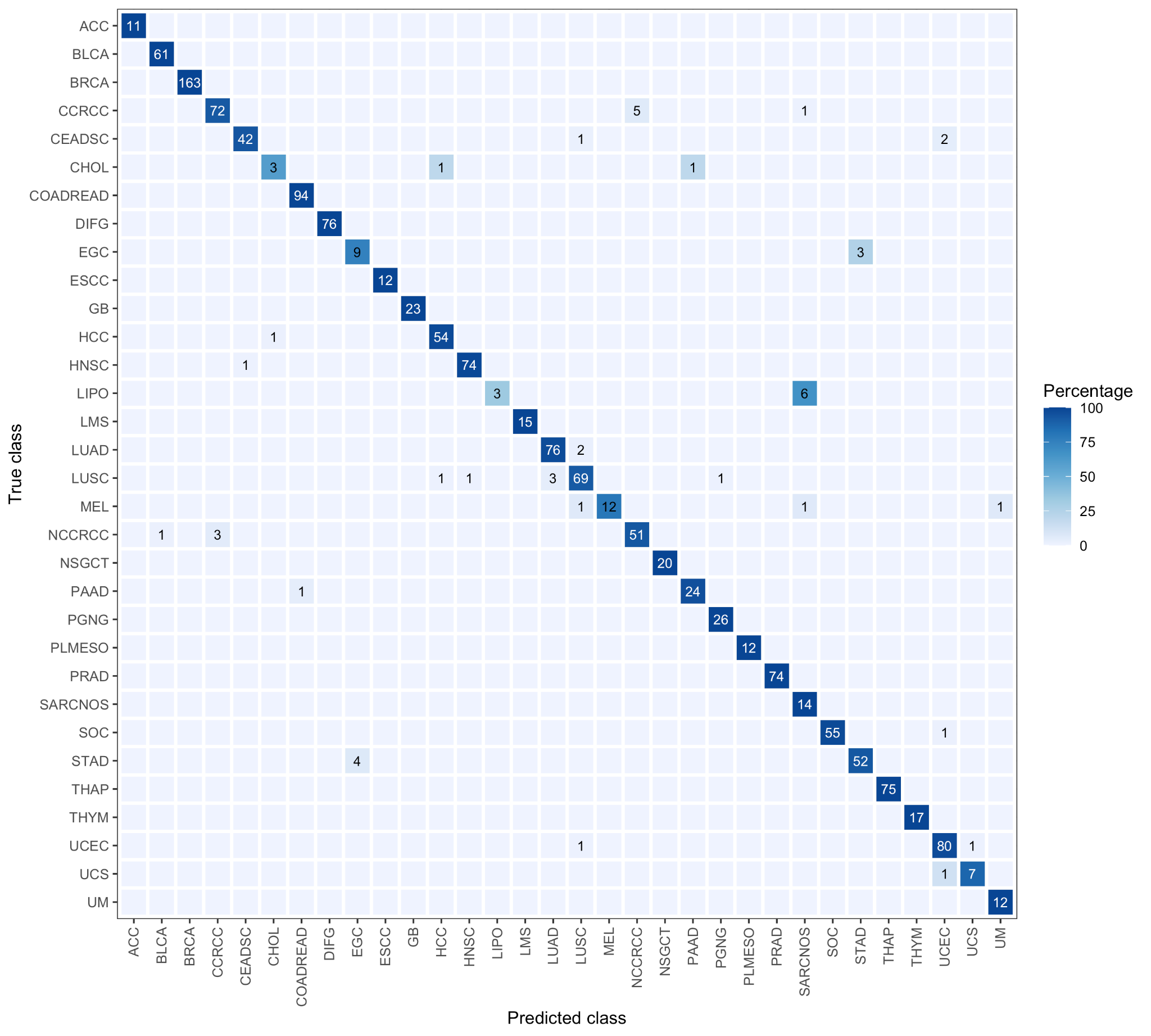


**Supplementary Figure 5. Confusion matrix of Ensemble predictions for IID validation data.** Each row corresponds to the true cancer type, and each column corresponds to the cancer type prediction. Coloured scale indicates the percentage of all samples in a particular class, while the numbers correspond to the number of samples predicted as a particular class.


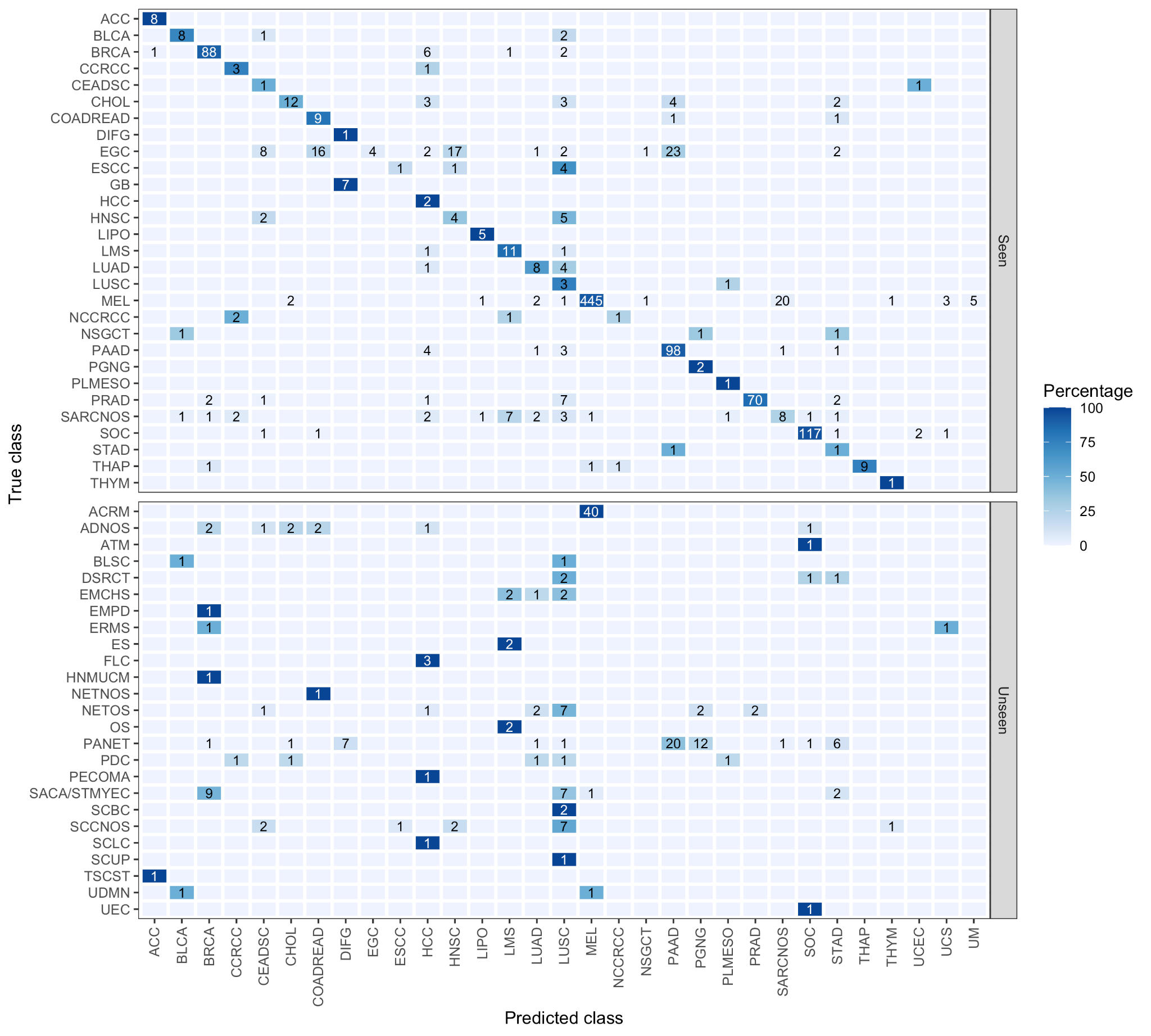


**Supplementary Figure 6. Confusion matrix of Resnet predictions for OOD test data.** Each row corresponds to the true cancer type, and each column corresponds to the cancer type prediction. Coloured scale indicates the percentage of all samples in a particular class, while the numbers correspond to the number of samples predicted as a particular class. Classes are grouped based on whether they were ‘Seen’ or ‘Unseen’ in the training data.


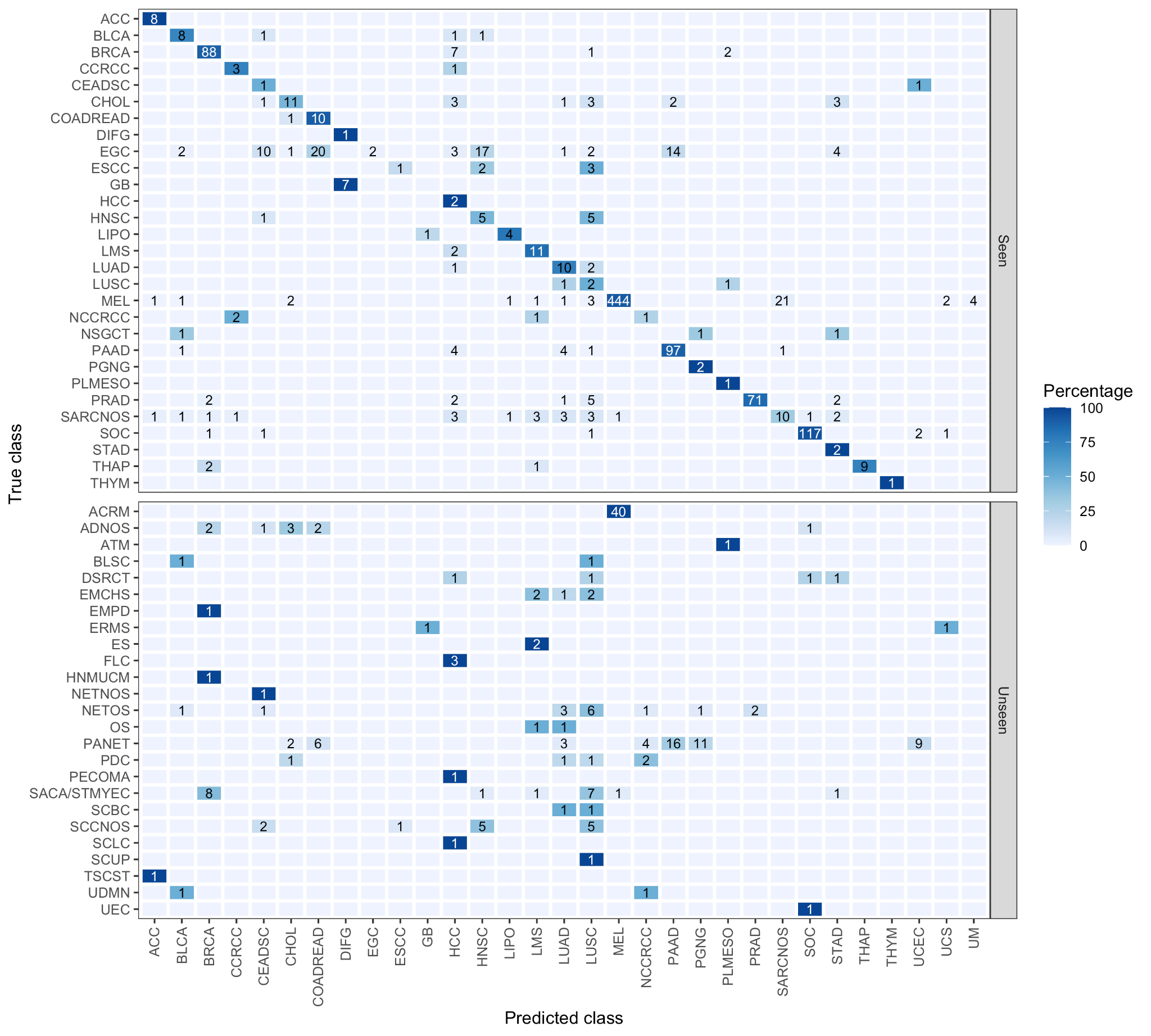


**Supplementary Figure 7. Confusion matrix of MCD predictions for OOD test data.** Each row corresponds to the true cancer type, and each column corresponds to the cancer type prediction. Coloured scale indicates the percentage of all samples in a particular class, while the numbers correspond to the number of samples predicted as a particular class. Classes are grouped based on whether they were ‘Seen’ or ‘Unseen’ in the training data.


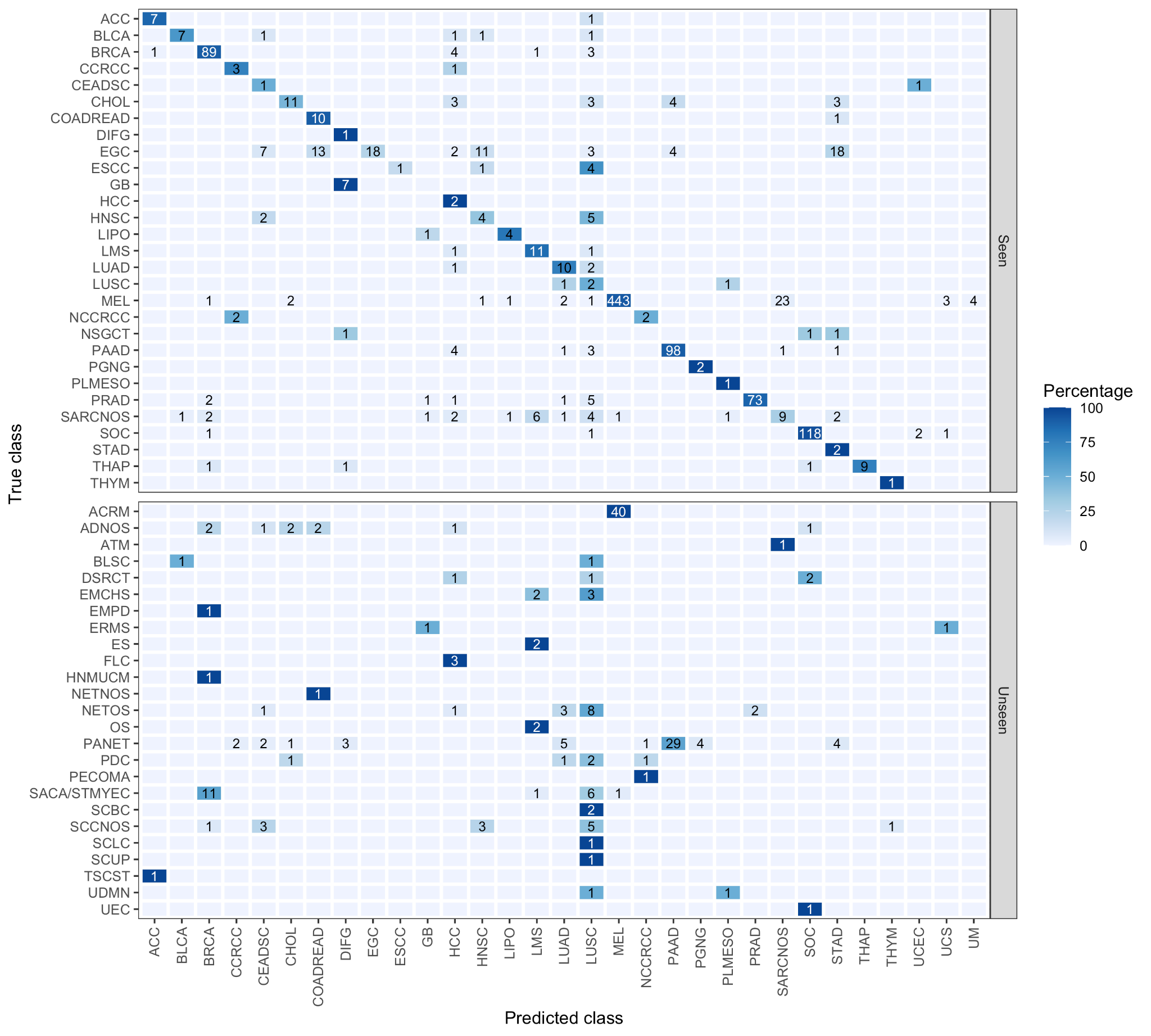


**Supplementary Figure 8. Confusion matrix of Bilipschitz predictions for OOD test data.** Each row corresponds to the true cancer type, and each column corresponds to the cancer type prediction. Coloured scale indicates the percentage of all samples in a particular class, while the numbers correspond to the number of samples predicted as a particular class. Classes are grouped based on whether they were ‘Seen’ or ‘Unseen’ in the training data.


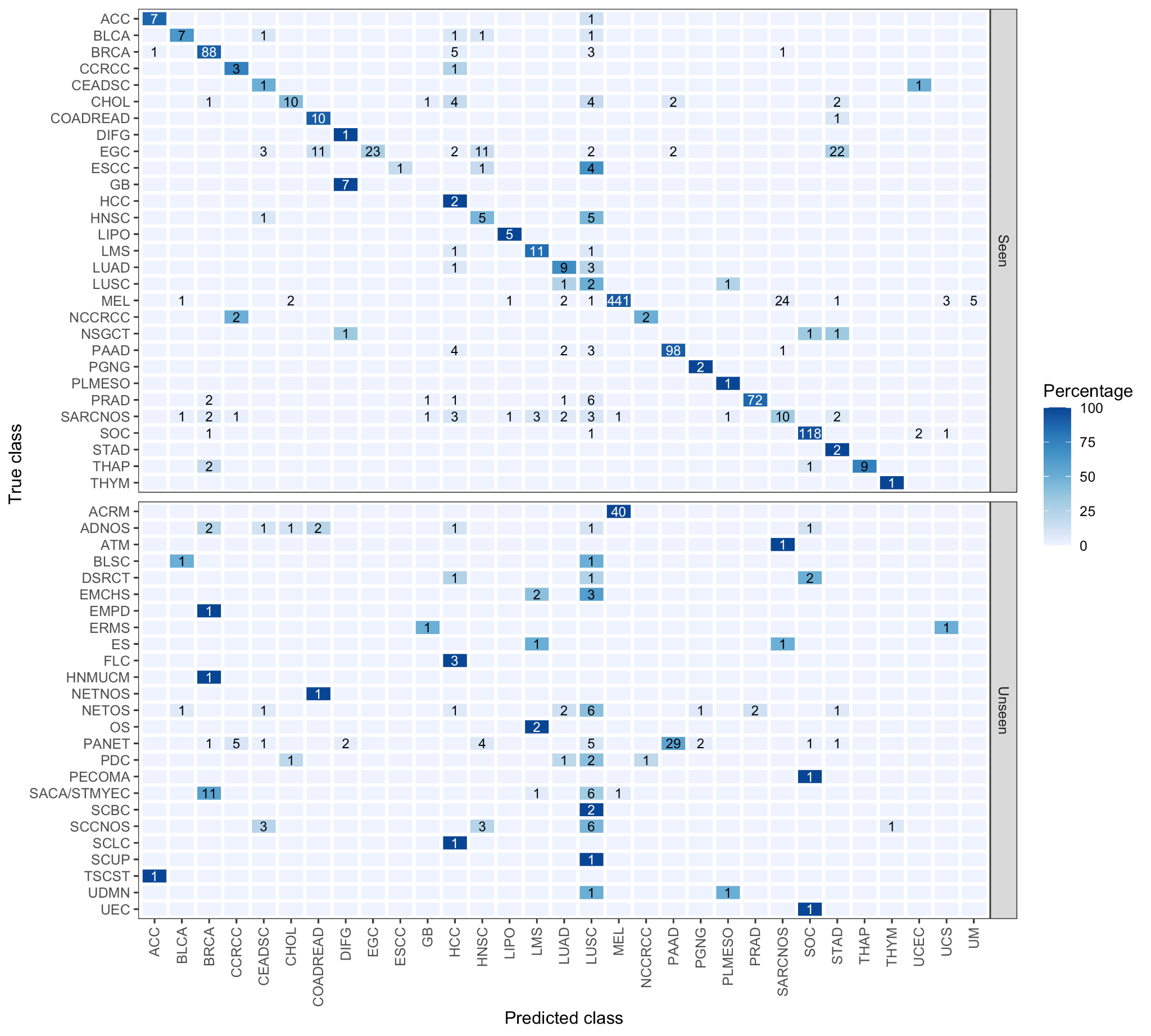


**Supplementary Figure 9. Confusion matrix of Ensemble predictions for OOD test data.** Each row corresponds to the true cancer type, and each column corresponds to the cancer type prediction. Coloured scale indicates the percentage of all samples in a particular class, while the numbers correspond to the number of samples predicted as a particular class. Classes are grouped based on whether they were ‘Seen’ or ‘Unseen’ in the training data.

**
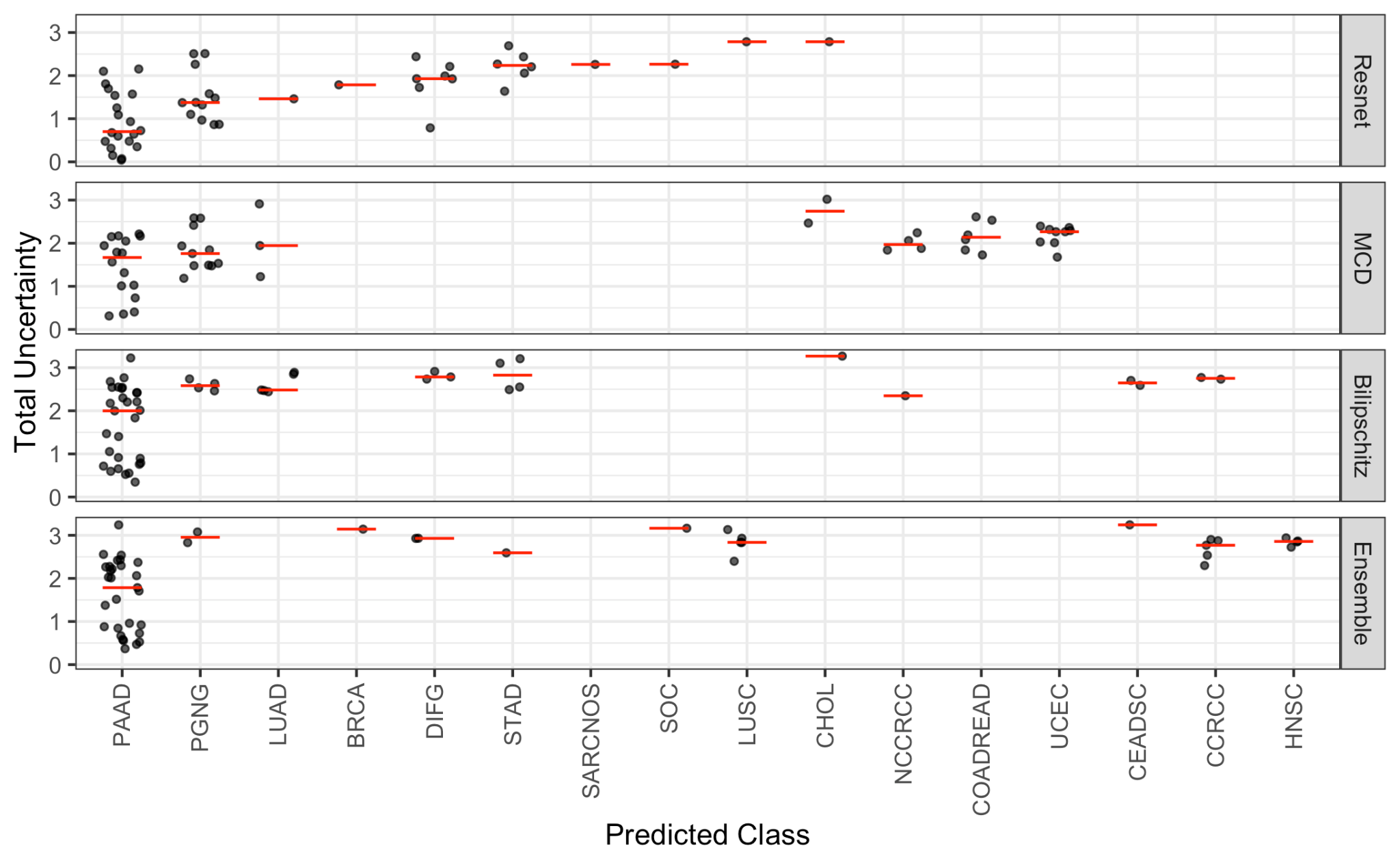
Supplementary Figure 10. Prediction uncertainties for the ‘unseen’ PANET class.** The horizontal red lines denoted median total uncertainty.


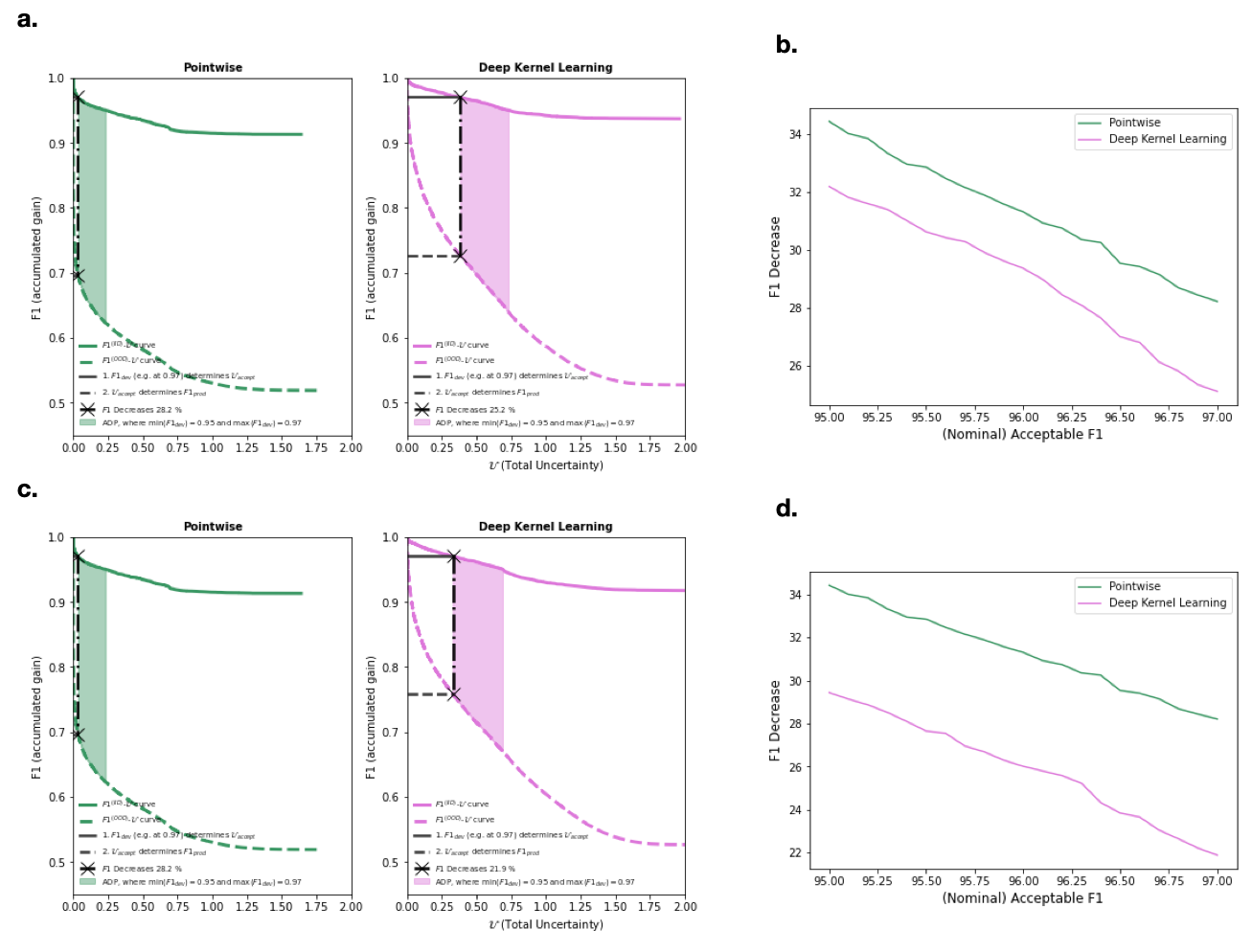


**Supplementary Figure 11. Quantifying ADP for CIFAR-10 (IID) and CIFAR-10-C (OOD) with Deep Kernel Learning.** **a** and **b** show results when the two models were trained for 200 epochs, allowing the Bayesian model to exceed the non-Bayesian model with CIFAR-10 validation accuracy. **c** and **d** show results when accuracy was controlled for to match the more poorly performing non-Bayesian model. **a** and **c** show the area between the F1-uncertainty curves for CIFAR-10 and CIFAR-10-C, respectively. Note that the shaded area between the curves is not proportional to ADP, since the plotted axis is uncertainty, and not the nominated acceptable F1-scores. The area under the lines in **b** and **d** are directly proportional to ADP metric.

# S4 Application of ADP to the CIFAR-10 dataset

As an additional experiment to demonstrate potential utility of ADP, the ADP metric was applied to the standard CIFAR-10 dataset, as well as the CIFAR-10-C (out-of-distribution) dataset. This was done by contrasting the non-Bayesian WideResnet model against a Bayesian deep kernel learning (DKL) model, which extended the WideResnet model by constructing a Gaussian Process at the final output layer. Both models had identical architecture and the DKL model did not use any bi-Lipschitz regularisation.

Both models were trained for 200 epochs on the popular CIFAR-10 training dataset. After training, CIFAR-10 test set accuracies were 91.29 % and 93.66 % for the non-Bayesian and Bayesian models, respectively. The ADP was calculated by deeming CIFAR-10 for the IID (i.e., ‘development’) dataset, and CIFAR-10-C for the OOD (i.e., ‘production’) dataset. ADP was calculated within the nominal development acceptable accuracy range of 95.00 % to 97.00 %. The resultant ADP was 31.27 % for the non-Bayesian model, and 28.93 % for the Bayesian model. As expected, the Bayesian model experienced less (Average) loss in accuracy when transitioning from (CIFAR-10) development to (CIFAR-10-C) production data.

Another iteration was repeated to control for accuracy. The DKL Bayesian model was re-trained with early stopping so that the CIFAR-10 (development) accuracies were both 91.3 %. The resultant ADP was 31.27 % for the non-Bayesian model, and 25.88 % for the Bayesian model. Hence the Bayesian model was far more robust to distribution shift, compared to the non-Bayesian model.

# S5 Supplementary Theory - Distributional Shift Model Effects

## S5.1 DL loss functions depend on IID data

DL models are often optimised by maximising the model’s likelihood, , of observing data [(Chicco and Jurman, 2020)](https://latex-staging.easygenerator.com/eqneditor/editor.php?latex=%5Cmathcal%7BD%7D%3D%5C%7B(%20y_i%2C%20%5Ctextbf%7Bx%7D_i)%5C%7D_%7Bi%3D1%7D%5EN#0), given model parameters [
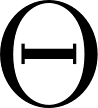
](https://www.codecogs.com/eqnedit.php?latex=%5CTheta#0). Under the Law of Total Probability, estimation of the likelihood becomes simple if data are assumed to be independent-and-identically-distributed (IID). Assuming IID makes the model likelihood equivalent to the product of individual sample likelihoods:

[
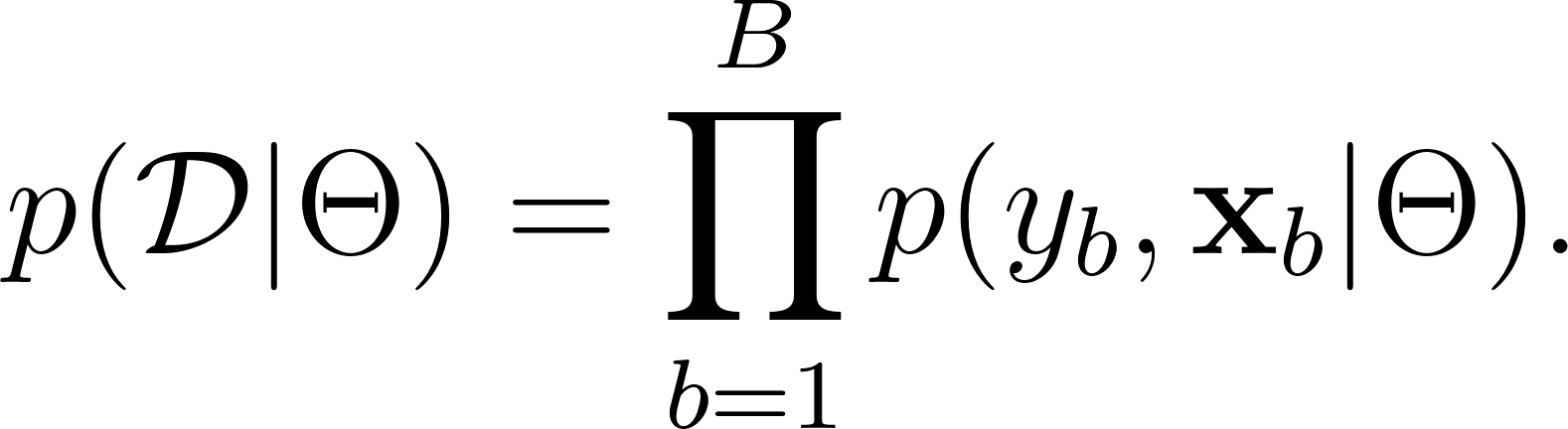
](https://www.codecogs.com/eqnedit.php?latex=p(%5Cmathcal%7BD%7D%7C%5CTheta)%20%3D%20%5Cprod_%7Bb%3D1%7D%5EBp(y_b%2C%20%5Ctextbf%7Bx%7D_b%7C%5CTheta).#0)

The individual sample likelihood is defined by [
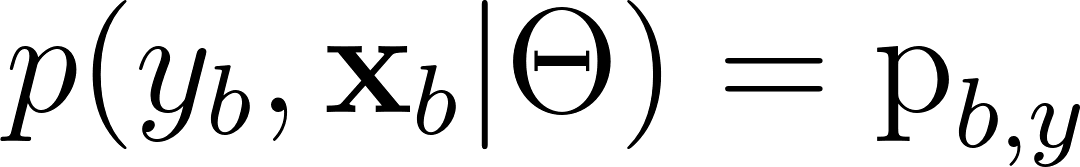
](https://www.codecogs.com/eqnedit.php?latex=p(y_b%2C%20%5Ctextbf%7Bx%7D_b%7C%5CTheta)%3D%5Ctext%7Bp%7D_%7Bb%2Cy%7D#0), where [
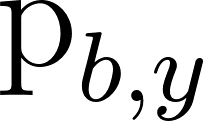
](https://www.codecogs.com/eqnedit.php?latex=%5Ctext%7Bp%7D_%7Bb%2Cy%7D#0) is the estimated probability corresponding to the correct label, [
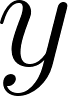
](https://www.codecogs.com/eqnedit.php?latex=y#0), from SoftMax output [
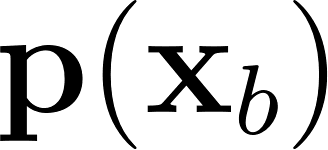
](https://www.codecogs.com/eqnedit.php?latex=%5Ctextbf%7Bp%7D(%5Ctextbf%7Bx%7D_b)#0). Without the IID assumption, the model likelihood would be, in most cases, intractable, or otherwise require more complex assumptions. This modelling assumption is, in large part, what makes generalisation difficult.

## S5.2 Out-of-domain data leads to ‘silent catastrophic failure’

A specific subtype of OOD data is when a sample, [
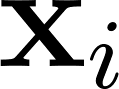
](https://www.codecogs.com/eqnedit.php?latex=%5Ctextbf%7Bx%7D_i#0), becomes *out-of-domain* [*
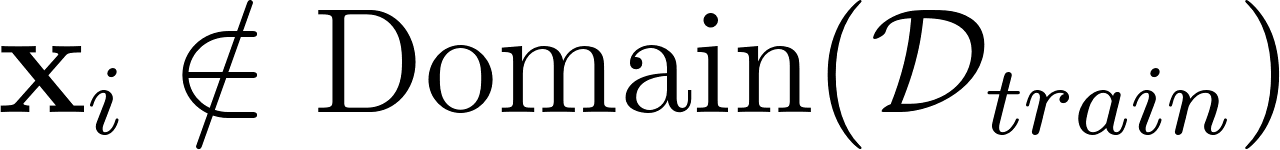
*](https://www.codecogs.com/eqnedit.php?latex=%5Ctextbf%7Bx%7D_i%20%5Cnotin%20%5Ctext%7BDomain%7D(%5Cmathcal%7BD%7D_%7Btrain%7D)#0), i.e. outside the training data’s support. This results in completely unreliable predictions and extreme overconfidence, which in production inference would amount to what we call ‘silent catastrophic failure’. Out-of-domain overconfidence in a parametric modelling context is referred to as ‘feature collapse’ or ‘posterior collapse’ in Bayesian DL literature [16], [17], ‘mode collapse’ in GAN literature [18], [19], and ‘no-overlap’ in causal inference literature [20]. In causal inference, out-of-domain data are more probable due to the paradox of not being able to observe counterfactual observations, hence the importance of accounting for out-of-domain sources of uncertainty.

# S6 Supplementary References

[1] K. Tomczak, P. Czerwińska, and M. Wiznerowicz, “The Cancer Genome Atlas (TCGA): an immeasurable source of knowledge,” *Contemp Oncol (Pozn)*, vol. 19, no. 1A, pp. A68-77, 2015, doi: 10.5114/wo.2014.47136.

[2] K. A. Hoadley *et al.*, “Cell-of-Origin Patterns Dominate the Molecular Classification of 10,000 Tumors from 33 Types of Cancer,” *Cell*, vol. 173, no. 2, pp. 291-304.e6, Apr. 2018, doi: 10.1016/j.cell.2018.03.022.

[3] D. R. Robinson *et al.*, “Integrative clinical genomics of metastatic cancer,” *Nature*, vol. 548, no. 7667, Art. no. 7667, Aug. 2017, doi: 10.1038/nature23306.

[4] S. Akgül *et al.*, “Intratumoural Heterogeneity Underlies Distinct Therapy Responses and Treatment Resistance in Glioblastoma,” *Cancers*, vol. 11, no. 2, Art. no. 2, Feb. 2019, doi: 10.3390/cancers11020190.

[5] L. G. Aoude *et al.*, “Radiomics Biomarkers Correlate with CD8 Expression and Predict Immune Signatures in Melanoma Patients,” *Molecular Cancer Research*, vol. 19, no. 6, pp. 950–956, Jun. 2021, doi: 10.1158/1541-7786.MCR-20-1038.

[6] P. Bailey *et al.*, “Genomic analyses identify molecular subtypes of pancreatic cancer,” *Nature*, vol. 531, no. 7592, Art. no. 7592, Mar. 2016, doi: 10.1038/nature16965.

[7] N. K. Hayward *et al.*, “Whole-genome landscapes of major melanoma subtypes,” *Nature*, vol. 545, no. 7653, Art. no. 7653, May 2017, doi: 10.1038/nature22071.

[8] J. H. Lee *et al.*, “Transcriptional downregulation of MHC class I and melanoma de- differentiation in resistance to PD-1 inhibition,” *Nat Commun*, vol. 11, no. 1, Art. no. 1, Apr. 2020, doi: 10.1038/s41467-020-15726-7.

[9] F. Newell *et al.*, “Multiomic profiling of checkpoint inhibitor-treated melanoma: Identifying predictors of response and resistance, and markers of biological discordance,” *Cancer Cell*, vol. 40, no. 1, pp. 88-102.e7, Jan. 2022, doi: 10.1016/j.ccell.2021.11.012.

[10] F. Newell *et al.*, “Whole-genome sequencing of acral melanoma reveals genomic complexity and diversity,” *Nat Commun*, vol. 11, no. 1, p. 5259, Oct. 2020, doi: 10.1038/s41467-020-18988-3.

[11] A.-M. Patch *et al.*, “Whole–genome characterization of chemoresistant ovarian cancer,” *Nature*, vol. 521, no. 7553, Art. no. 7553, May 2015, doi: 10.1038/nature14410.

[12] A. Scarpa *et al.*, “Whole-genome landscape of pancreatic neuroendocrine tumours,” *Nature*, vol. 543, no. 7643, Art. no. 7643, Mar. 2017, doi: 10.1038/nature21063.

[13] R. Kundra *et al.*, “OncoTree: A Cancer Classification System for Precision Oncology,” *JCO Clinical Cancer Informatics*, Feb. 2021, doi: 10.1200/CCI.20.00108.

[14] D. Chicco and G. Jurman, “The advantages of the Matthews correlation coefficient (MCC) over F1 score and accuracy in binary classification evaluation,” *BMC Genomics*, vol. 21, no. 1, p. 6, Jan. 2020, doi: 10.1186/s12864-019-6413-7.

[15] Y. Wang, H. Huang, C. Rudin, and Y. Shaposhnik, “Understanding How Dimension Reduction Tools Work: An Empirical Approach to Deciphering t-SNE, UMAP, TriMAP, and PaCMAP for Data Visualization.” arXiv, Aug. 24, 2021. doi: 10.48550/arXiv.2012.04456.

[16] J. Lucas, G. Tucker, R. Grosse, and M. Norouzi, “Understanding Posterior Collapse in Generative Latent Variable Models,” Jul. 2019, Accessed: Jun. 30, 2022. [Online]. Available: https://openreview.net/forum?id=r1xaVLUYuE

[17] J. van Amersfoort, L. Smith, A. Jesson, O. Key, and Y. Gal, “On Feature Collapse and Deep Kernel Learning for Single Forward Pass Uncertainty,” arXiv, arXiv:2102.11409, Mar. 2022. doi: 10.48550/arXiv.2102.11409.

[18] J. Li, A. Madry, J. Peebles, and L. Schmidt, “On the Limitations of First-Order Approximation in GAN Dynamics,” in *Proceedings of the 35th International Conference on Machine Learning*, Jul. 2018, pp. 3005–3013. Accessed: May 31, 2022. [Online]. Available: https://proceedings.mlr.press/v80/li18d.html

[19] Y. Saatci and A. G. Wilson, “Bayesian GAN,” in *Advances in Neural Information Processing Systems*, 2017, vol. 30. Accessed: May 31, 2022. [Online]. Available: https://proceedings.neurips.cc/paper/2017/hash/312351bff07989769097660a56395065-Abstract.html

[20] A. Jesson, S. Mindermann, U. Shalit, and Y. Gal, “Identifying Causal-Effect Inference Failure with Uncertainty-Aware Models,” in *Advances in Neural Information Processing Systems*, 2020, vol. 33, pp. 11637–11649. Accessed: Jun. 30, 2022. [Online]. Available: https://proceedings.neurips.cc/paper/2020/hash/860b37e28ec7ba614f00f9246949561d-Abstract.html
